# Polyphenols bind G4-Quadruplex structures and act as epigenetic modifiers with anti-cancer effects

**DOI:** 10.17179/excli2025-8507

**Published:** 2025-08-01

**Authors:** Marco Antonio Meraz-Rodriguez, Manuel Humberto Cháirez-Ramírez, Karen Griselda de la Cruz-López, Rubén Francisco González-Laredo, Alejandro García-Carrancá

**Affiliations:** 1 Biomedical Cancer Research Unit, Institute for Biomedical Research, National Autonomous University of Mexico (UNAM) & National Cancer Institute, Mexico City, Mexico (Unidad de Investigación Biomédica en Cáncer, Instituto de Investigaciones Biomédicas, Universidad Nacional Autónoma de México & Instituto Nacional de Cancerología, Ciudad de México, México); 2 PhD Program in Biochemical Sciences, Institute for Biomedical Research, National Autonomous University of Mexico (UNAM), Mexico City, Mexico (Programa de Doctorado en Ciencias Bioquímicas, Instituto de Investigaciones Biomédicas, Universidad Nacional Autónoma de México (UNAM), Ciudad de México, México); 3 PhD Program in Biomedical Sciences, Institute for Biomedical Research, National Autonomous University of Mexico (UNAM), Mexico City, Mexico (Programa de Doctorado en Ciencias Biomédicas, Instituto de Investigaciones Biomédicas, Universidad Nacional Autónoma de México (UNAM), Ciudad de México, México); 4 National Laboratory for the Evaluation of Biotic Products (LaNAEPBi), Service Unit, National Technological Institute of Mexico/Durango Institute of Technology, Functional Foods and Nutraceuticals Research Group, Durango, Mexico (Laboratorio Nacional de Apoyo a la Evaluación de Productos Bióticos (LaNAEPBi), Unidad de Servicio, Tecnológico Nacional de México/I.T. de Durango, Grupo de Investigación de Alimentos Funcionales y Nutracéuticos, Durango, México)

**Keywords:** G4 quadruplex, G4 ligands, dietary polyphenols, epigenetic regulation, gene expression, anti-cancer molecules

## Abstract

G4-quadruplexes (G4s) are non-canonical structures of nucleic acids that develop in guanine rich regions of DNA and RNA. Due to their presence in oncogenic promoters and telomeres, G4s represent attractive targets in anticancer drug designs. G4s have also been the subject of recent research regarding their role as epigenetic modulators, supporting their participation in epigenetic processes that control gene expression. The development of small compounds that preferentially target G4s have led to a better understanding of how G4s control these mechanisms. Natural products have greatly contributed to the development of many successful examples of compounds with excellent anticancer activities. Therefore, it is important to investigate ligands targeting G4-quadruplexes in natural products such as dietary polyphenols and their derivatives. In this review, we provide an overview of the latest research on natural compounds, with especial emphasis on dietary polyphenols, as G4-quadruplex targeted ligands. We also discuss dietary polyphenols' structural chemistry that could facilitate their characterization as G4 ligands, highlighting their potential in the development of anticancer drugs. Finally, we explore polyphenols' potential mechanisms of action in regulating epigenetic machinery through G4 binding, thereby providing insights for the development of safe and effective therapeutical tools against cancer.

## Introduction

The human genome's sequencing revealed that over 50 % of its sequence consists of repetitive sequences that were once believed to be merely the results of genetic evolutionary forces but are now understood to have crucial roles in biology, including the regulation of chromatin structure, gene expression, DNA replication, and genomic organization. Repeating sequences can fold into alternative DNA structures other than the right-handed DNA double helix, or B-DNA form (Wang and Vasquez, 2014[184]). Most genomic DNA is structured in B-DNA, the most thermodynamically stable configuration. However, at least 15 non-canonical DNA configurations have been described, and up to 13 % of the human genome may be organized into these alternative structures (Guiblet et al., 2018[63]). G4 quadruplexes (G4s) belong to these non-canonical structures which are formed in a dynamic process depending on the cellular context (Marshall et al., 2020[106]). G4 quadruplexes are DNA and RNA secondary structures formed by self-association of guanine bases forming stacked G-tetrads that are stabilized by Hoogsteen hydrogen bonding and connected by stretches of nucleotides, or loops, that vary in length and composition (Varshney et al., 2020[182]). Recent scientific interest in G4s has increased due to their importance in biological processes such as DNA replication, gene expression, telomere maintenance, and cell death (Bochman et al., 2012[19]; Awadasseid et al., 2021[8]). Epigenetic modifications are reversible alterations of nucleic acids that do not change their chromosomal DNA sequence, and are connected to many disorders, including cancer (Esteller, 2008[50]). Thus, chemical agents that revert aberrant epigenetic alterations are potentially promising therapeutic tools. These compounds have been called epigenetic drugs or “epidrugs” (Montalvo-Casimiro et al., 2020[116]). G4 structures are connected to several epigenetic mechanisms and have been identified as structural targets of small aromatic compounds or ligands (Mukherjee et al., 2019[118]). In fact, many G4 binding ligands with the ability to regulate gene expression have been obtained from natural sources (Sengupta et al., 2019[155]). These G4 stabilizing agents have been shown to drastically reduce oncogene expression levels both *in vitro* and *in vivo* (Awadasseid et al., 2021[8]). Therefore, G4 ligands ought to be considered in the future development of therapeutic interventions, especially in cancer treatment. Dietary polyphenols are widely recognized for their numerous beneficial effects on human health (Tsao, 2010[177]). Polyphenols regulate epigenetic pathways and have potent anti-cancer activities by modulating genes involved in cell transformation, tumor growth, angiogenesis, and metastasis (Cháirez-Ramírez et al., 2021[25]). These aromatic compounds chemically interact with and stabilize G4 quadruplexes; therefore, understanding the relationship between dietary polyphenols and G4s can further expand the molecular mechanisms by which polyphenols exert their anti-cancer effects (Bag et al., 2023[9]). In this review, we will discuss novel key regulatory elements of the genome, G4 quadruplexes, their role in cancer biology, gene expression control, and the interaction of different dietary polyphenols with these structures. This will highlight the importance of studying polyphenols as novel stabilizing ligands of G4 quadruplexes from an epigenetic perspective. We emphasize on chemical nature, composition, and regulatory functions of polyphenols and their derivatives to promote G4 quadruplex-targeted anti-cancer drug development.

### Structural characteristics of G4 quadruplexes and their presence in biological environments

**Figure 1:**
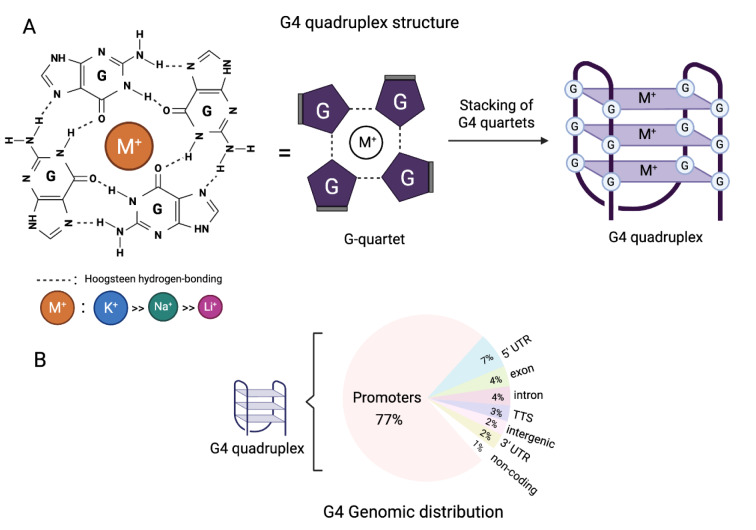
Structure of G-quadruplex (G4). (A) Hoogsteen base pairing and a central cation (M+) stabilize a guanine tetrad; monovalent cations are preferred in the following order: potassium (K+) > sodium (Na+) > lithium (Li+). (B) Genomic distribution of G4 quadruplex

The idea that G4 structures form in the context of genomic DNA was first considered when the crystal structure of a telomeric G4 was reported for the first time two decades ago (Parkinson et al., 2002[128]). Since then, biophysical experiments have shown that many guanine rich DNA and RNA sequences fold into G4 and have laid the foundations for predicting G4 structure formation (Burge et al., 2006[22]). G4s have been recognized as true cell features through a variety of computational sequence analyses and studies that have identified G4s in cellular genomes using chemical, molecular, and imaging techniques (Varshney et al., 2020[182]). G4 quadruplexes (G4s) develop under conditions of physiological activity in single-stranded DNA and RNA G-rich sequences (Lipps and Rhodes, 2009[100]). To form a G4 quadruplex (G4), G-quartets must first be formed with four guanines (G) binding via Hoogsteen base pairing to create a G-quartet. Each guanine in a G-quartet serves as both donor and acceptor of two hydrogen bonds, which hold the four guanines together (Figure 1(Fig. 1)). By stacking at least two G-quartets together, aromatic G-quartet interactions produce the formation of a G4 quadruplex (G4) (Choi and Majima, 2011[31]). The G4 quadruplex DNA's architecture is impacted by the negatively charged core channel in the quadruplex, which chelates cations such as K+, Na+, and Li+. Potassium cations (K+) especially aid in the development and stability of G4 by reducing the repulsion between the oxygen atoms in the central cavity due to its bigger size compared to Na+ and Li+ (Sen and Gilbert, 1990[154]). Potassium is a better coordinator atom due to its lower dehydration energy and a higher intracellular concentration (~140 mM) compared to Na+ (5-15 mM) (Harrell, 2006[68]) (Figure 1(Fig. 1)). The formula GxNyGxNyGxNyGx generally describes the nucleic acid sequence that has the capacity to generate G4 structures, where x = 2 guanosine residues (or more) and y = 1-7 nucleotides (N). G4s can form intramolecularly from a single strand of nucleic acid or intermolecularly from several strands both in the context of DNA and RNA (Meier-Stephenson, 2022[108]). While basic sequence relationships exist between G4s, their true nature is a wide family of structures where the length and orientation of the intervening loops between G tracts directly control their folded geometry and thermodynamic stability (Burge et al., 2006[22]). DNA G4s can exhibit several different configurations, but RNA structures often take a predominantly parallel form (Joachimi et al., 2009[80]). The ribose sugar's 2′-hydroxyl group in RNA creates steric limitations, restricting the topology of RNA G4s exclusively to the parallel conformation. In fact, it's been hypothesized that G-rich RNA sequences are more likely to form quadruplex structures than DNA since RNA molecules lack a complementary strand. Nonetheless, most of the thousands of mammalian RNA sequences that could fold into G4s *in vitro* are unfolded in cells, most likely due to cellular machinery that unfolds RNA G4s globally (Guo and Bartel, 2016[64]). Over 700,000 G4s have been biophysically mapped in purified human genomic DNA by high-throughput sequencing, but the presence of a G4 motif in the genome alone does not guarantee that G4 structures exist at these locations *in vivo*; rather, it only suggests that a G4 may form there and that their presence require experimental demonstration with the use of techniques such as immunofluorescence and chromatin immunoprecipitation sequencing with a G4-specific antibody. G4 structures have been found to be particularly abundant in certain parts of the genome, including promoters, telomeres, and transcription factor binding sites, rather than being randomly distributed across the genome (Chambers et al., 2015[26]) (Figure 1(Fig. 1)). The first G4 structure was found in the single-stranded DNA overhangs of repeating human telomeric motifs (Bochman et al., 2012[19]). Cellular senescence regulation, abnormal telomere processing, and the initiation and progression of cancer have all been linked to telomeric G4s. Therefore, molecules that interact with telomeric intramolecular G4s have been proposed as potentially effective anticancer agents (Kosiol et al., 2021[90]). Computer simulations have shown that over 40 % of gene promoter regions can form G4 quadruplexes, particularly those controlling genes related to cell proliferation, survival, and differentiation (Li et al., 2024[98]). The existence of G4 quadruplexes in the promoter regions of MYC, KRAS, BCL2, and other oncogenes has been demonstrated to hinder their expression and have an inhibitory effect on cell proliferation and in the development of tumors. These oncogenic promoter G-quadruplex structures may serve as regulatory points in the development of cancer, making G4 quadruplex structures promising therapeutic targets in the field of cancer research (Li et al., 2024[98]). Finally, RNA G4s are most frequently found in UTRs, but they can also appear in coding sequences. G4s can be also found in microRNAs (miRNAs) and long noncoding RNAs (lncRNAs), as well as in their target locations, suggesting that G4s may influence the interaction of miRNAs and lncRNAs with their target mRNAs (Kwok et al., 2016[92]). Human ribosomal RNA has also been reported to form exposed G4s on the ribosomal surface, implying that G4s may also have a role in the recruitment of non-ribosomal proteins and/or polysome assembly (Mestre-Fos et al., 2019[109]).

## G4 Quadruplexes and Their Role in Cancer

Cancer is a large group of diseases characterized by uncontrolled cell proliferation, driven by transformed cells that undergo evolution through natural selection (Brown et al., 2023[21]). The prevalence of cancer as a fatal disease is evident in its annual global mortality rate of 10 million individuals with a worldwide economic burden surpassing 1 trillion dollars annually. For cancer to initiate, an extended number of cell divisions, an accumulation of oncogenic genetic and epigenetic mutations, and a permissive tissue environment unable to prevent the division and persistence of increasingly abnormal cells are required (Brown et al., 2023[21]). The genetic and epigenetic changes that occur in cancer have been described as Hanahan and Weinberg's "Hallmarks of Cancer" (Hanahan, 2022[66]). These hallmarks include sustaining proliferation, evasion of growth suppressors, resistance to cell death, induction of angiogenesis, activation of invasion and metastasis, deregulation of cellular energetics, evasion of immune destruction and genomic instability (Hanahan, 2022[66]). Cancer is largely driven by the accumulation of genetic abnormalities and genomic instability. G4s, through their potential impact on genomic stability, may play a role in promoting cancer development (Richl et al., 2024[143]). As tumors progress, the intracellular chemical conditions are considerably altered. Indeed, tumors possess different dielectric properties in comparison to normal cells. Such changes can be recognized in aggressive cancer cells, which have been found to overexpress potassium channels. Changes in intracellular K+ ion concentration heavily affect G4 quadruplex stabilities, which in turn can modulate gene expression during tumor progression (Tateishi-Karimata et al., 2018[170]). Due to their prevalence in many cancer-related genes, G4 structures have become attractive targets for cancer therapy (Figueiredo et al., 2023[53]). Notably, there is a global enrichment of G4s in tumors compared to non-cancerous tissues. This increased prevalence of G4 structures may contribute to the activation of transcriptional programs that promote cell proliferation during cancer development. Importantly, cancer cells do not remain epigenetically static, but rather undergo continuous evolution, resulting in dynamic changes in gene expression, as well as modifications in DNA structures like G4s. This epigenetic plasticity in cancer results in the development of drug resistance, which is lethal for patients (Robinson et al., 2025[145]).

### Oncogene promoter regions and G4s

Cancer arises from mutations in proto-oncogenes, tumor-suppressor, and DNA-repair genes (Dakal et al., 2024[37]). Oncogenes can be described as a modified version of proto-oncogenes, genes involved in normal cell division and growth, with deleterious mutations (Dakal et al., 2024[37]). Therefore, an oncogene is formed when a proto-oncogene is altered to produce an excessive amount of its copies or to increase its activity levels. Consequently, cell growth control is lost due to defects in different regulatory systems, which translates into altered cell behavior and uncontrolled proliferation of cancer cells (Dakal et al., 2024). Over 20,000 genes feature G4 quadruplex motifs in their promoter sequences. Interestingly, these have been found to be especially over-represented in cancer-promoting genes such as *c-Myc*, *c-KIT*, *hTERT*, *K-RAS*, *BCL2* and *VEGF* (Burger et al., 2005[23]; Cogoi and Xodo, 2006[34], 2016[33]; Palumbo et al., 2008[125], 2009[126]; Ramsay and Gonda, 2008[138]; Bell et al., 2011[13]; Biffi et al., 2014[18]; Salvati et al., 2014[150]; Zorzan et al., 2016[201]; Jana et al., 2017[77]; Cheng et al., 2019[30]; Ducani et al., 2019[47]; Hänsel-Hertsch et al., 2020[67]; Prasad et al., 2020[136]; Sheikh et al., 2022[157]; Bokhari and Hamar, 2023[20]; Kaloni et al., 2023[81]; Ash et al., 2024[6]; Mondal et al., 2024[113]; Table 1(Tab. 1); References in Table 1: Ash et al., 2024[6]; Bell et al., 2011[13]; Bokhari and Hamar, 2023[20]; Burger et al., 2005[23]; Chaudhuri et al., 2021[27]; Cheng et al., 2019[30]; Cogoi and Xodo, 2006[34], 2016[33]; Dang, 2012[38]; Dhanasekaran et al., 2023[42]; Fekete et al., 2012[52]; González et al., 2009[62]; Kaloni et al., 2023[81]; Mondal et al., 2024[113]; Palumbo et al., 2008[125]; Prasad et al., 2020[136]; Ramsay and Gonda, 2008[138]; Salvati et al., 2014[150]; Sheikh et al., 2022[157]; Simonsson et al., 1998[160]; Zorzan et al., 2016[201]). These oncogenes share in their promoter region a nuclease hypersensitive element (NHE). These regions are guanine rich, thus allowing the formation of non-B-DNA conformation such as G4. Interestingly, it has been shown that G4s formed by promoter sequences are involved in the regulation of gene expression and are crucial for cancer development. Directing our focus towards G4s located in oncogene promoter regions has the potential to be an efficient strategy for fighting cancer, but further investigation is needed to fully understand its vast possibilities (Monsen, 2023[115]).

### G4 quadruplexes in tumors

**Figure 2:**
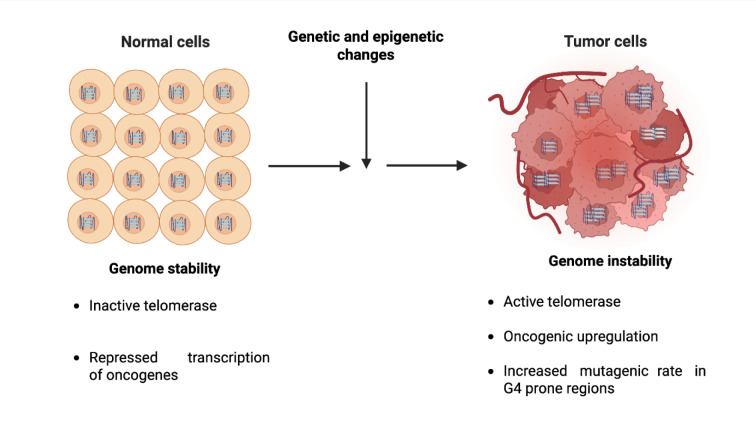
Tumor progression and G4. During tumor progression, genetic and epigenetic changes in tumor cell increase G4 quadruplex abundance, creating genomic instability in tumors.

The variation in response and resistance to anticancer therapy is attributed to the presence of heterogeneity within and between tumors. Thus, the variations in the presence and abundance of G4 structures in tumors are now being explored as potential biomarkers for cancer prognosis. G4 quadruplex maps generated from patient-derived tumors have shown that G4s are differentially enriched between tumors and associated with highly expressed gene promoters. Depending on the G4 landscape, tumors can be stratified into G4-based subtypes, suggesting the coexistence of multiple G4 states within tumors. Tumor cells with greater G4 levels are more susceptible to treatment by molecules that specifically target G4s, which highlights G4s as genomic features with potential for future diagnostics and therapeutics (Hänsel-Hertsch et al., 2020[67]) (Figure 2(Fig. 2)). Antibodies and fluorescent probes that selectively target G4 structures have also been employed to visually identify G4s in cancers. Samples from hepatocellular carcinoma and intrahepatic cholangiocarcinoma exhibited a significantly higher number of BG4-positive nuclei, compared to non-neoplastic tissue with an increase in BG4-positive staining in metastases. Additionally, an increase of BG4-positive nuclei in stomach adenocarcinoma and signet ring cell carcinoma compared to non-neoplastic tissue sampled from the same patient was reported. These results suggest that G4 differences might be dependent on alterations of cellular processes that regulate genome stability or changes in the chromatin state at G4 quadruplex sites *in situ* (Biffi et al., 2014[18]). The difference in G4 content could be a key factor in distinguishing between normal and tumor cells. G4 fluorescent detection performed on serum samples obtained from patients diagnosed with colorectal cancer also revealed a significant increase in G4 levels among individuals with colorectal cancer in comparison with healthy individuals. Therefore, G4 serum detection could be considered as a novel biomarker for colorectal cancer diagnosis (Zhang et al., 2024[198]).

## Role of G4 Quadruplex Structures in Epigenetic Mechanisms

Epigenetics, reversible chemical modifications of DNA, RNA and histone proteins, regulate chromatin functions without changing the DNA sequence and allow an organism to develop and adapt to environmental changes (Montalvo-Casimiro et al., 2020[116]). Gene expression changes depending on cellular phenotype or function in response to different stimuli, such as developmental stages, cellular differentiation, or tissue-specific cell lineages. Epigenetics create a regulatory complex layer that links genomic sequences to actionable mutations highlighting the importance of this regulatory system and revealing that epigenetic alterations are some of the main mechanisms underlying many human diseases, such as cancer, which arises from aberrant genetic and epigenetic alterations, which play a key role in malignant transformation, tumor progression, and prognosis. Thus, epigenetics' complex mechanisms and their significance in disease development must be understood for cancer treatment (Montalvo-Casimiro et al., 2020[116]). The epigenetic machinery consists primarily of three interrelated components that operate together in the organization of chromatin and gene expression at the molecular level. These components include DNA methylation, histone post-translational modifications, and regulatory non-coding RNAs (ncRNAs) (Roberti et al., 2019[144]; Zhang et al., 2024[198]). The different patterns of histones and DNA chemical modifications form “the epigenetic code”-a complex interaction of epigenetic components with positive and negative feedback mechanisms that control gene expression (Reina and Cavalieri, 2020[141]). G4s definitively fall into these criteria, as distinctive genomic regions that dynamically adopt interconverting structural conformations that can affect gene expression favorably or negatively, causing transcriptomic alterations due to their genomic localization (Reina and Cavalieri, 2020[141]). G4 quadruplex structures and DNA epigenetic alterations often coexist, establishing a connection between G4s and epigenetic processes which have been observed to be dysregulated in cancer (Montalvo-Casimiro et al., 2020[116]).

### DNA epigenetic modifications and G4 quadruplexes

**Figure 3:**
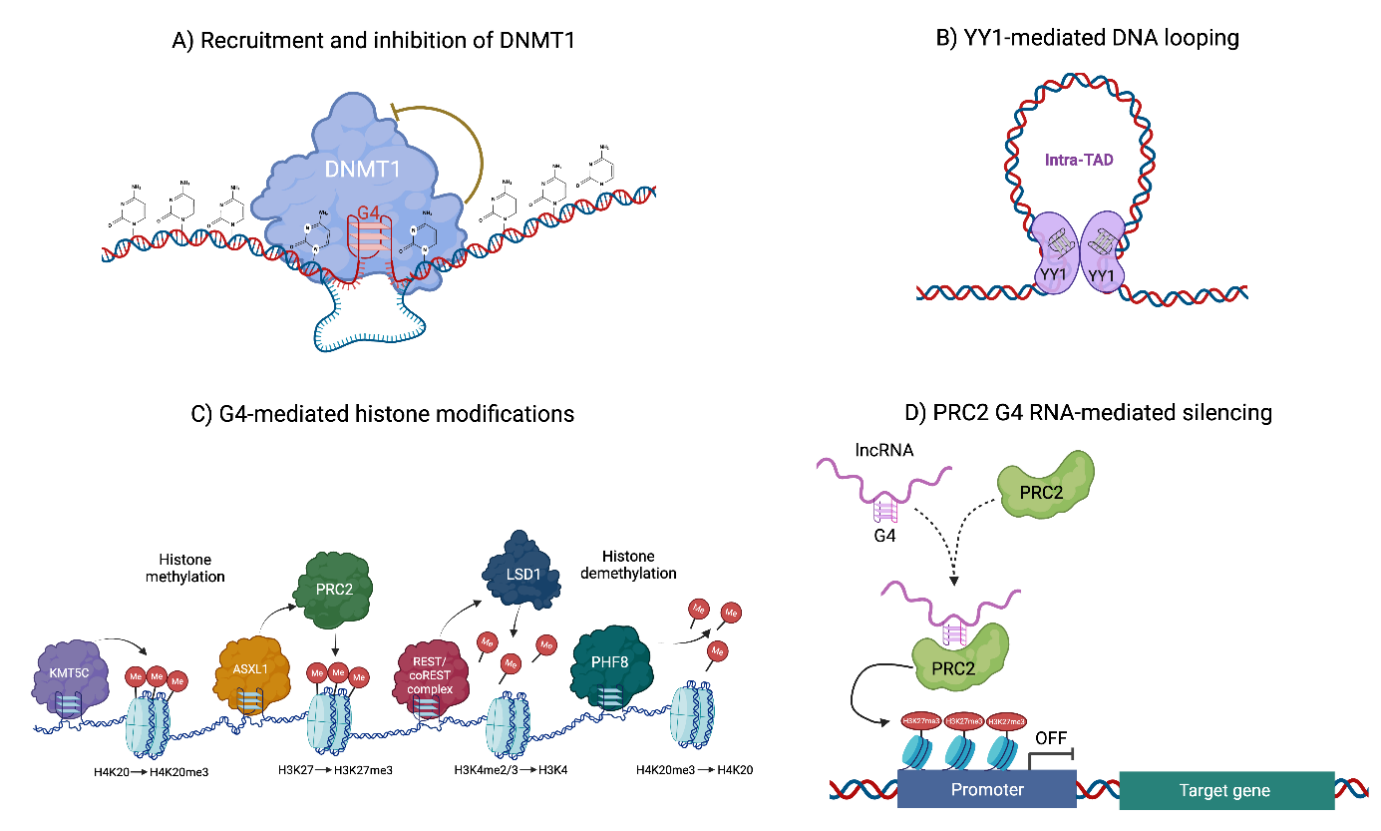
Epigenetic functions of G4 quadruplexes. G4 quadruplexes exert their epigenetic regulation through interaction with epigenetic machinery. (A) Recruitment and inhibition of DNMT1, (B) G4-YY1 mediated DNA looping, (C) G4-mediated histone post-translational modifications, and (D) PRC2-G4 mediated RNA silencing.

DNA methylation of cytosine's carbon 5 (5-methylcytosine) is an important epigenetic mark for development and illness. In mammals, the development-essential DNA methyltransferases DNMT1, DNMT3A, and DNMT3B install and maintain CpG dinucleotide cytosine methylation (Deaton and Bird, 2011[40]). Most tissues have stable methylation patterns, however during crucial cellular events, methylation can be dynamic at certain loci to affect gene expression (Reik et al., 2001[140]). Tumor cells are characterized by global DNA demethylation and local hypermethylation (Kisseljova and Kisseljov, 2005[87]). Tumor-specific DNA hypermethylation patterns of gene regulatory elements of tumor suppressor genes cause transcriptional inactivation and are a hallmark of cancer (Hanahan, 2022[66]). Alterations of hypermethylated patterns in repetitive sequences activate transposable elements and cause chromosomal instability, which are also linked to carcinogenesis and metastasis (Miranda Furtado et al., 2019[111]). Conversely, gene body demethylation allows transcriptional activation at numerous erroneous locations and consequently stimulating proto-oncogene gene expression (Portela and Esteller, 2010[135]). G4 quadruplexes and DNA methylation coexist since both G4s and hypomethylated CGIs are linked with actively transcribed genes (Mao et al., 2018[104]). DNMT1 installs methylation and has a high binding affinity and selectivity for G4 DNA structures; therefore, its abundance at G4 regions without methylation was surprising. G4 DNA prevents DNA methylation by recruiting and inhibiting DNMT1, uncovering a novel and unexpected feature of G4 structures as epigenomic features that promote an unmethylated state and have a role in the epigenome formation (Mao et al., 2018[104]) (Figure 3(Fig. 3)). 

### Histone post-translational modifications and G4 quadruplexes

Covalent post-translational modifications of histones are another epigenetic machinery axis linked to DNA accessibility and gene expression. Incorporating reversible chemical modifications to the amino- or carboxy-terminal domains of histone proteins have various effects on genomic structure and output (Peterson and Laniel, 2004[133]). The main modifications of histone proteins include: sumoylation, ribosylation, phosphorylation, acetylation, ubiquitylation, and methylation; of these, acetylation and methylation are the most prevalent and well-studied, and they typically take place close to enhancer and promoter genomic regions (Wang et al., 2009[189]). Each covalent modification is added and removed by specialized enzymes. While histone methyltransferases (HMTs) and demethylases (HDMs) coordinate histone methylation, histone acetyltransferases (HATs) and deacetylases (HDACs) regulate histone acetylation (Portela and Esteller, 2010[135]). Abnormal histone post-translational modifications alter gene expression and cause human diseases due to their role in gene regulation and cellular function. Understanding the reversibility of these marks is crucial for treating disorders characterized by epigenome dysregulation such as cancer (Montalvo-Casimiro et al., 2020[116]). G4 structures serve as binding locations for effector protein complexes that modify histones. The histone methyltransferase KMT5C can attach to both RNA and DNA G4 structures found in telomeric chromatin. Specifically, KMT5C modifies the lysine 20 residue of nucleosomal histone H4 by adding three methyl groups (H4K20me3), which promotes the condensation of chromatin (Takahama et al., 2013[169]). In contrast, the histone demethylase PHF8, which specifically targets H4K20, has been linked to promoters with G4 structures of highly expressed genes that are found in open chromatin regions (Hou et al., 2019[70]). Another important example is the REST/coREST repressor complex, which transports the histone H3K4-specific demethylase LSD1 to certain chromatin sites that contain G4 structures, such as the p21 and hTERT gene promoters (Hussain et al., 2017[73]; Saha et al., 2017[149]). In the RNA context, mammalian PRC2 binds thousands of RNA transcripts in vivo, but prefers G4 RNA quadruplexes. This enrichment at Polycomb target genes allows RNA-mediated gene control in cis. G4 structures evict PRC2 from the nucleosome and restrict its methyltransferase activity, regulating its occupancy at target genes temporarily (Wang et al., 2017[187]; Beltran et al., 2019[14]) (Figure 3(Fig. 3)).

### Nuclear architecture and G4 quadruplexes

Chromatin is hierarchically folded in larger chromosomal loop structures called topologically associated domains, or TADs. TADs are the genomic structure and function units that define the regulatory expression patterns (Du et al., 2021[45]). Dysfunction of TADs and aberrant TAD border fusion can lead to many developmental disorders and illnesses. Numerous studies suggest that TAD boundary disintegration in cancer cells leads to aberrant oncogene activation, indicating a link between TAD folding and transcriptional aberrations in cancer (Du et al., 2021[45]). G4s engage with architectural proteins that affect nucleosome placement and three-dimensional chromatin organization. In humans, non-nucleosomal DNA carries G4-forming sequences which often correspond with TAD borders (Reina and Cavalieri, 2020[141]). Loop anchors at the borders of TADs are linked by the protein CTCF, a highly conserved protein and critical regulator of genome architecture and gene expression. CTCF, together with cohesin, insulate large chromatin loop domains from each other (Du et al., 2021[45]). Within TADs, Yin Yang 1 (YY1), a key mediator in cell proliferation and death, mediates enhancer-promoter interactions, analogous to CTCF-mediated DNA looping (Verheul et al., 2020[183]). Depletion of YY1 binding sites abolishes enhancer-promoter interactions and gene expression (Weintraub et al., 2017[190]). G4s colocalize with CTCF at multiple genomic locations and increase CTCF binding to its DNA consensus sequence in vitro (Lyu et al., 2022[103]). Moreover, G4 stabilization increases CTCF binding and chromatin loop formation, showing that G4 structures are important for CTCF-mediated long-range genomic interactions (Wulfridge et al., 2023[192]). YY1 was also discovered to be a protein that interacts with G4 structures, with a significant overlap between YY1-binding sites and G4 structures (Li et al., 2021[95]). In contrast to CTCF, YY1 directly binds to G4 quadruplexes. The dimerization of YY1 and its interaction with G4 structures participate in YY1-induced DNA looping as YY1 displacement from G4 sites significantly impairs intra-domain interactions. Furthermore, the administration of G4-stabilizing ligands not only affects the expression of genes that have G4 structures at their promoters but also impacts the expression of genes that are linked with distal G4 structures and brought closer together through YY1-mediated DNA looping (Li et al., 2021[95]) (Figure 3(Fig. 3)).

## G4 Quadruplex Stabilizing Ligands

G4 quadruplexes exist in living cells and play a critical role in controlling gene expression and other processes making these structures novel targets for drug design (Tian et al., 2018[175]). The development of ligands that are capable of binding and stabilizing G4 structures may not only facilitate the characterization of these structures *in vivo* but also contribute to the development of new therapeutic and diagnostic strategies (Summers et al., 2021[165]). Many G4 ligands interact in a relatively indiscriminate manner, recognizing G4s of diverse topologies, thereby demonstrating their potential as multi-targeting agents (Figueiredo et al., 2024[54]). These molecules influence cancer cell growth by interacting with G4s and their effects include interference with telomere function, stabilization of G4 in the promoters of oncogenes, post-translational gene regulation by targeting mRNA 5′-untranslated regions, impeding helicase unwinding, induction of genomic instability, and the modulation of the epigenetic machinery to control gene expression. G4 polymorphism provides the opportunity to search and develop compounds that can identify a single G4 topology (Figueiredo et al., 2024[54]). Telomerase is a reverse transcriptase that adds repeated segments to the 3'-end of telomeric DNA and is abundantly expressed in most cancers (Hanahan, 2022[66]). In fact, the initial motivation behind the creation of these innovative therapeutic approaches was the notion that these molecules would bind to telomeric ends and form persistent, liganded G4 structures preventing telomeric ends from being accessed by telomerase for extension (Sun et al., 1997[166]). Nevertheless, studies revealed rapid antitumoral effects that were not consistent with telomerase suppression, which typically requires a prolonged period and multiple cell divisions before inducing cell death (Iachettini et al., 2024[74]). Thus, the antitumoral potential of G4 ligands was further expanded to include G4s found in gene promoters. Ever since, numerous G4-targeted ligands have been identified that can regulate the activity of genes containing a sequence capable of generating a G4 structure in their promoters (Kim, 2019[86]). Stabilized G4s located in promoter regions could impede the movement of RNA polymerases, thus preventing gene transcription, or serve as sites for the recruitment of transcription factors, thereby facilitating gene transcription (Kim, 2019[86]). Currently, there have been limited research investigations that have examined alterations in gene expression on a comprehensive scale across the entire genome (Marchetti et al., 2009[105]). This could be achieved by utilizing techniques that allow the comprehensive identification of ligand binding sites in the natural chromatin environment (Spiegel et al., 2020[162]). Currently, the G-Quadruplex Ligands Database (http://www.g4ldb.com) has over 3200 compounds that specifically target G4 structures (Wang et al., 2022[188]). Targeting G-quadruplex DNA poses a significant scientific challenge due to its great polymorphism and relatively low abundance compared to canonical duplex DNA. G4 quadruplex-stabilization involves π-π stacking and electrostatic interactions, which leads to the binding of a ligand to the G-quartet found on the outer surface of the quadruplex (Hud and Plavec, 2006[72]). In contrast, both the groove and the backbone phosphates can interact with G4 ligands without the need for a flat aromatic structure. Hence, molecules that exhibit decreased planarity and establish interactions with the grooves and/or backbone phosphates may be advantageous for selectively targeting G4s. 

The primary challenge in designing compounds that specifically interact with G4 DNA is to create big, planar aromatic structures that can effectively stack with a G quartet platform, while still maintaining sufficient solubility in water. They must display both hydrophobic and hydrophilic properties (Monchaud and Teulade-Fichou, 2008[112]). One way to achieve this duality is by including protonable sidearms, such as amine groups, surrounding the aromatic core that make the molecule water-soluble, with the charges located far from the hydrophobic center (Sun et al., 1997[166]). G4 ligands have a higher aromatic ring count, positive charges, and number of hydrogen bond donors than what would be considered optimal for favorable pharmacokinetic qualities (Spiegel et al., 2020[162]). In fact, no G4 ligand has progressed past Phase II trials due to low pharmacological qualities (Santos et al., 2021[152]). Thus far, the only ligand that has progressed to this stage is the fluoroquinolone derivative, quarfloxin (CX-3543) which specifically binds to G4s found in ribosomal DNA and interferes with the binding of nucleolin (Drygin et al., 2009[44]). CX-5461 is another G4 ligand that is now undergoing advanced phase I clinical studies for patients with BRCA1/2 defective malignancies (Xu et al., 2017[193]). Nevertheless, some G4 ligands have demonstrated their effectiveness in human cancer tumor xenografts (Marchetti et al., 2009[105]). Performing structure-activity relationship studies on G4 ligands, focusing on physicochemical qualities such as planarity, polarity, lipophilicity, and rotatable bonds, would allow for achieving balance between G4 binding, solubility, and permeability (Spiegel et al., 2020[162]). The main challenge is to balance selectivity and affinity, even if multiple or genome wide G4 targeting strategies might work (Santos et al., 2021[152]). The small molecule BRACO-19 has been found to downregulate the expression of hTERT, leading to the inhibition of telomerase activity and resulting in the shortening of telomere length (Burger et al., 2005[23]). Additionally, the cationic porphyrin TMPyP4 has been shown to downregulate the expression of *c-Myc*, an oncogene that is over-expressed in many tumor cells (Thumpati et al., 2025[173]). Studies into the structure of G4 complexes with natural macrocyclic molecules, such as telomestatin and its derivatives, which completely cover the outer G4 quartet in the telomeric G4, revealed significant p-stacking and electrostatic interactions (Wang et al., 2024[186]). Indeed, large macrocyclic molecules often exhibit strong attraction to most G4 DNA configurations, making it difficult to selectively bind to a single G4. Contrary to macrocyclic molecules, small crescent-shaped G4 ligands can stack over the outer G-tetrad by recruiting neighboring residues and forming significant π-π and electrostatic interactions (Wang et al., 2024[186]). The primary obstacle in the pursuit of developing drugs that target G4 structures is achieving G4 selectivity. G4 quadruplexes share a compacted G quartet core surrounded by multiple loops. Consequently, these compounds face challenges in differentiating between various G4 structures, particularly those with similar topologies (Wang et al., 2024[186]). The lack of selectivity of G4 ligands, which are both found in normal and malignant cells, is the main obstacle in the clinical implementation of these compounds as anticancer treatments (Iachettini et al., 2024[74]). Conducting structure-activity relationship studies could greatly enhance the physicochemical properties of ligands and facilitate the process of ligand design and development to achieve specificity and selectivity while maintaining high affinity (Asamitsu et al., 2019[5]).

### G4 quadruplex destabilizing ligands

Molecular interactions and stabilization of G4s in cells have been studied for over two decades in order to understand their mechanisms. These discoveries have led to rethought G4-targeting strategies to find molecular tools to unfold G4s due to their prevalence in the human genome and transcriptome (now referred to as the G4ome) and their involvement in human disorders. This has been hindered by the lack of standard assays and techniques to consistently assess G4 destabilization, unlike G4 stabilization. Only a few number of compounds have exhibited such a feature, some of which are disputed. These chemicals' unwinding capacities have been tested *in vitro* with assays designed for single studies. Therefore, there is still no consensus on their cellular relevance (Lejault et al., 2021[94]).

## Dietary Polyphenols and Their Biological Activities

The polyphenols´ structure comprises multiple hydroxyl groups (-OH) attached to a carbon atom within an aromatic ring, allowing to combine with various functional groups to form esters, ethers, and carbon-carbon bonds, leading to a vast number of different structural arrangements (Liu et al., 2024[101]), including phenolic acids (hydroxybenzoic and hydroxycinnamic acids), stilbenes, lignans, flavonoids (flavanols, isoflavones, anthocyanins, flavanones, flavones, and flavonols), and tannins (condensed and hydrolyzable). More than 50,000 identified polyphenols (Tsao, 2010[177]) and ~8000 compounds have been found and characterized in food and medicinal plants (flavonoids represent ~75 % of total polyphenols in dietary sources). Cancer is influenced by complex physiological and environmental factors, with diet playing a crucial role. Western diets (high in unhealthy fats and refined carbohydrates) are associated with an increased cancer risk. In contrast, diets rich in fruits and vegetables (~1,193 ± 510 mg/day polyphenols intake) have been linked to cancer prevention (Li et al., 2023[96]; Adolph and Tilg, 2024[1]). Polyphenols exert their protective effects through various mechanisms, including epigenetic modulation, which has gained significant attention for its potential in cancer prevention (Silva et al., 2019[159]). Despite the increase in cancer cure rate in recent times (~67 % in adults), the use of synthetic drugs faces significant challenges, including financial burdens and potential side effects in patients, which can affect life quality or even lead to death (van den Boogaard et al., 2022[181]). Numerous studies have suggested that polyphenols are linked to a lower risk of cancer with fewer side effects (Sharma et al., 2022[156]).

### Therapeutic potential of polyphenols in cancer treatment

**Figure 4:**
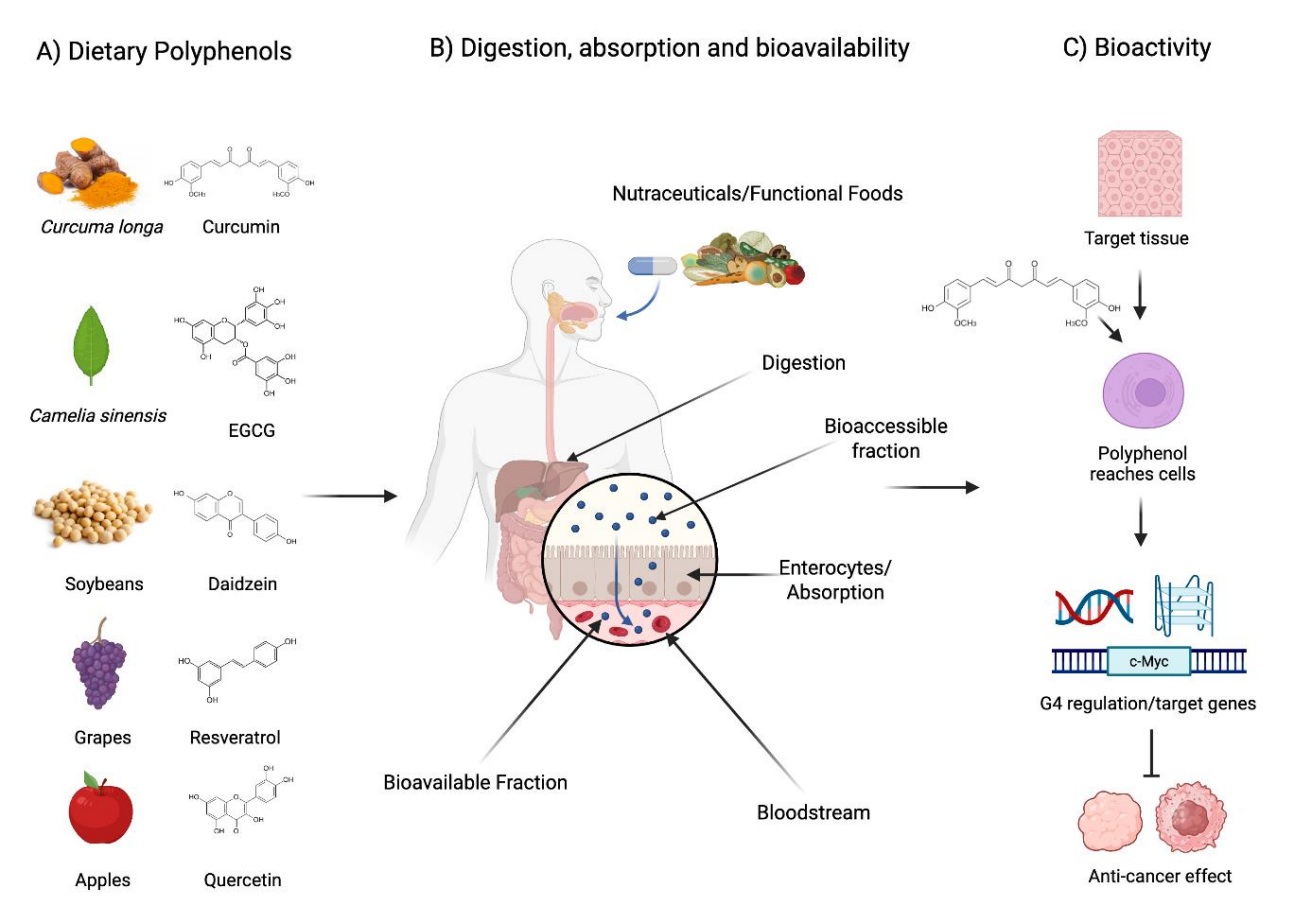
The Complex Pathway of Dietary Polyphenols: From the Diet to Target Cells. A) Functional foods and nutraceuticals are polyphenols-rich sources. B) Polyphenols are ingested and subjected to different processes in the gastrointestinal tract, including absorption, metabolism, and systemic bioavailability. C) Polyphenols reach target tissues and accumulate within the cells, modifying the G4s at oncogenes, leading to epigenetic regulation and anticancer effects.

Polyphenols are key players involved in the prevention and treatment of cancer due to their versatility for modulating multiple biological processes, including, but not limited to antioxidant properties, signaling pathways modulation, promotion of programmed cell death, cell cycle arrest prevention angiogenesis, and regulation of epigenetic mechanisms. These mechanisms are related to tumor development and progression (Figure 4(Fig. 4)). One key process through which polyphenols exhibit their anticancer properties is prompting apoptosis. Compounds like quercetin and epicatechin have been found to trigger the release of cytochrome c, activate caspases, and elevate the expression of death receptors D4/D5; thereby increasing the vulnerability of cancer cells to apoptosis via the extrinsic pathway (Srivastava et al., 2016[163]; Pereyra-Vergara et al., 2020[132]). Kaempferol has been shown to arrest the cell cycle at the G2/M phase through the ATM/CHEK2/KNL1 pathway in hepatocellular cancer (Li et al., 2024[97]). Polyphenols can also modulate cell growth. Resveratrol has shown noteworthy anti-cancer properties against gastric cancer by promoting programmed cell death and inhibiting cancer cell growth via the PI3K/Akt/p53 signaling pathway (Dong et al., 2024[43]). Curcumin in turn has been found to suppress the proliferation of HeLa cells by influencing both the NF-κB and Wnt/β-catenin pathways and arrest of the cell cycle at G2/M, leading to sub-G1 apoptosis when used in conjunction with 5-Fluorouracil (Ghasemi et al., 2019[59]). Polyphenols neutralize free radicals and reactive oxygen species (ROS), which may cause DNA damage, mutations, and tumor progression. EGCG decreases the production of ROS triggered by miR483-3p in a dose-dependent manner, thereby reducing the metastatic capabilities in liver cancer (Kang et al., 2021[83]). Despite the extensive examples of the anti-proliferative, anti-tumor, and anti-apoptotic effects of polyphenols, the precise cellular and molecular mechanisms that underlie their activity remain unclear. The flavonoid structure satisfies the molecular structure of G4 ligands with a flat chromophore backbone with an additional carboxyl group that facilitates charge transfer. This structure may effectively insert itself into the planar scaffold formed by G-tetrads. Therefore, the flavonoid structure has been explored for its potential role as an anticancer therapy by affecting the stability of G4-quadruplexes found on oncogene promoters. Additionally, dietary polyphenols that have been shown to bind to G4-quadruplexes using spectroscopic and biophysical methods *in vitro* including circular dichroism spectroscopy, UV fluorescence titration, computational studies like molecular docking, chemical structural analysis, and NMR. 

## Polyphenols as G4 Quadruplex Stabilizers

**Figure 5:**
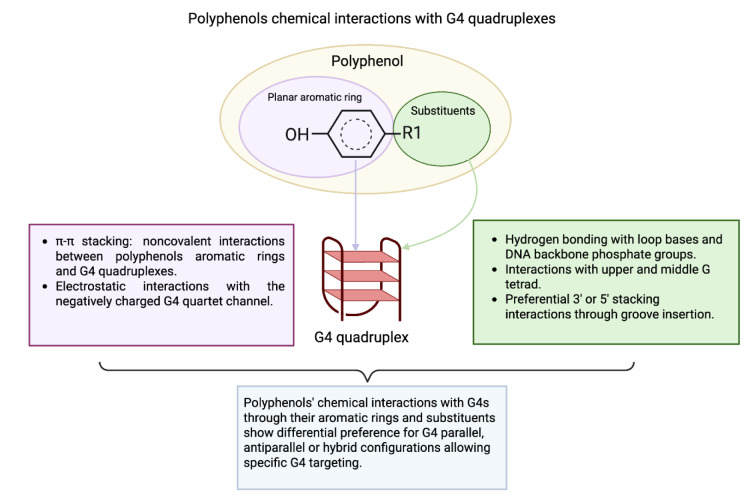
Chemical interactions between polyphenols and G4 quadruplexes. Polyphenols interact with G4 quadruplexes through their aromatic rings and their substituents. Aromatic rings interact with G4 quadruplexes through π-π stacking and electrostatic interactions. In contrast, substituents interact with the loops and grooves of the structure through different mechanisms, such as hydrogen bonding and preferential stacking with the 5' or 3' end. These interactions make polyphenols G4 binders with high selectivity for specific G4 (parallel, antiparallel, or hybrid) configurations.

Polyphenols have been related to multiple anticancer benefits such as tumor growth reduction, fewer side effects, and efficacy enhancement of therapies like chemotherapy and radiation. However, to date, their full potential has not been fully explored. Polyphenols are increasingly being recognized as an alternative for concomitant therapies and as chemosensitizers because of their diversity and accessibility, with higher biocompatibility and lower systemic toxicity when used in moderate doses (Duda-Chodak and Tarko, 2023[48]; Jakobušić Brala et al., 2023[76]). A promising objective is to incorporate them into current cancer therapies, as they enhance the sensitivity of cancer cells. G4-quadruplexes (G4s) promote recombination events and mutations essential for cancer progression. Polyphenols can inhibit transcription and telomere elongation in cancerous cells, making them crucial targets for research focused on therapeutic applications. Investigating the binding properties of naturally occurring compounds that interact with G4s, like dietary polyphenols, is highly beneficial for drug discovery, particularly regarding their selectivity for polymorphic G4 configurations (Bag et al., 2023[9]). When compared to most G4 ligands, polyphenols have fewer aromatic cores, and their configuration (phenolic rings connected through a styrene double bond and many -OH groups) promotes hydrogen bonding with G4 loops and grooves instead of stacking, as demonstrated in laboratory studies and simulations (Platella et al., 2020[134]). This interaction with G4 grooves and loops could increase selectivity and lower toxicity *in vivo* compared to stacker ligands (Platella et al., 2020[134]) (Figure 5(Fig. 5)). Polyphenols are also well-suited for further modification to enhance targeting via chemical analysis and the custom design of G4 ligands (Ye et al., 2024[195]). Polyphenols must penetrate the cellular membrane to reach the nucleus and bind with promoter G4 quadruplexes to modulate gene expression. Complexation assays have demonstrated nuclear fluorescence of four flavonoids-azaleatin, quercetin, fisetin, and morin-in neuroblastoma cells, suggesting effective cellular uptake (De et al., 2022[39]). Curcumin exhibited strong nuclear fluorescence in SF-767 glioma cells (Ghosh and Ryan, 2014[60]) while galangin and kaempferol displayed higher nuclear autofluorescence in mouse hepatocellular carcinoma Hepa-1c1c7 cells (Mukai et al., 2009[117]). Quercetin accumulates in the nucleus and mitochondria of HepG2 cells through an active mechanism independent of nuclear pore transport, thereby affecting transcription (Notas et al., 2012[122]). Curcumin, extracted from the rhizome of *Curcuma longa*, is a prominent polyphenol with notable G-quadruplex (G4) binding capabilities, as demonstrated in Table 2(Tab. 2) (References in Table 2: AL Zahrani et al., 2020[3]; Anand David et al., 2016[4]; Ashrafizadeh et al., 2020[7]; Bag et al., 2023[9]; Bai et al., 2013[11], 2021[10]; Balaga et al., 2023[12]; Bhattacharjee et al., 2016[16], 2017[17], 2018[15]; Calderon-Montano et al., 2011[24]; Chen et al., 2021[29]; Dabeek and Marra, 2019[36]; De et al., 2022[39]; Delmas et al., 2011[41]; Du et al., 2013[46]; Dwivedi et al., 2021[49]; Franceschin et al., 2014[55]; Franciosoa et al., 2014[56]; Garcia-Canton et al., 2012[58]; Ghosh and Ryan, 2014[60]; Goh et al., 2022[61]; Imran et al., 2021[75]; Jakobušić Brala et al., 2023[76]; Jha et al., 2016[79]; Khojasteh et al., 2014[84]; Kong et al., 2021[89]; Krasieva et al., 2015[91]; Lakhanpal and Rai, 2007[93]; Mikutis et al., 2013[110]; Mondal et al., 2016[114]; Mukai et al., 2009[117]; Neveu et al., 2010[121]; Notas et al., 2012[122]; Obeng et al., 2020[123]; Pandya et al., 2021[127]; Pattanayak et al., 2016[129]; Paul et al., 2019[131]; Platella et al., 2021[134]; Rajput et al., 2021[137]; Ribaudo et al., 2022[142]; Rocca et al., 2024[146]; Roy et al., 2022[147], 2023[148]; Sanchez-Martin et al., 2022[151]; Sarao et al., 2021[153]; Shen et al., 2022[158]; Soriano-Lerma et al., 2024[161]; Stężycka and Frańska, 2023[164]; Sun et al., 2006[167], 2007[168]; Tawani and Kumar, 2015[171]; Tawani et al., 2017[172]; Tian and Liu, 2020[174]; Touil et al., 2011[176]; Tyagi et al., 2020[179]; Wang et al., 2015[185]; Wright et al., 2013[191]; Zenkov et al., 2022[197]; Zhang et al., 2009[199]; Zhao et al., 2023[200]). Studies have demonstrated its ability to stabilize G4 structures in the promoters of KRAS and c-Myc, leading to a reduction in oncogene expression and promoting cytotoxicity in metastatic breast cancer cells. At the same time, synthetic derivatives such as Cur-4 and dimethylcurcumin enhance these effects by increasing binding affinity and targeting mechanisms, promoting the degradation of the androgen receptor in prostate cancer (Jha et al., 2016[79]; Pattanayak et al., 2016[129]; Dwivedi et al., 2021[49]; Pandya et al., 2021[127]; Roy et al., 2022[147]). Quercetin, a flavonoid present in citrus fruits, onions, and several other foods, can decrease *c-Myc* expression by as much as 50 % in cancer cell lines, while rutin, the glycosylated form of quercetin, enhances the stability of the G4-polyphenol complex, offering an additional benefit (Sun et al., 2006[167]; Lakhanpal and Rai, 2007[93]; Tawani and Kumar, 2015[171]; Anand David et al., 2016[4]; Bhattacharjee et al., 2017[17]; Tawani et al., 2017[172]; Tyagi et al., 2020[179]; Zenkov et al., 2022[197]; Bag et al., 2023[9]). Indeed, rutin, which is derived from *Styphnolobium japonicum*, exhibits selective binding to *c-Myc* and telomeric G4s (see Table 2(Tab. 2)) (Sun et al., 2007[168]; Ribaudo et al., 2022[142]; Stężycka and Frańska, 2023[164]). Likewise, kaempferol, which is abundant in green leafy vegetables and herbs, is more effective than its isomer morin, present in mulberries, figs, and other plants, in stabilizing *c-Myc* and **VEGF** G4s, achieving reductions in *c-Myc* expression of up to 77 % in malignant cells, due to their differing molecular structures (Calderon-Montano et al., 2011[24]; Bhattacharjee et al., 2018[15]; Dabeek and Marra, 2019[36]; Paul et al., 2019[131]; Rajput et al., 2021[137]; Zenkov et al., 2022[197]; Balaga et al., 2023[12]). Resveratrol, derived from grapes and red wine, along with its derivatives, polydatin and viniferin, engage with *c-Myc* and telomeric G4s, demonstrating effects that hinder the proliferation of melanoma cells, with the dimeric form of viniferin exhibiting a greater G4 affinity (see Table 2(Tab. 2)) (Tian and Liu, 2020[174]; Platella et al., 2021[134]). In contrast, naringenin, commonly found in citrus fruits, tends to bind to duplex DNA over G4, whereas fisetin, which is found in strawberries and nuts, prefers G4 binding, highlighting how structural differences affect their interactions (Touil et al., 2011[176]; Bhattacharjee et al., 2016[16]). A wider range of polyphenols-including rosmarinic acid from rosemary, luteolin from celery, genistein and daidzein from legumes, gallic acid from tea and fruits, myricetin from berries, EGCG from green tea, and xanthones from tricyclic structures-also interact with G4s, often reducing oncogene activity or hindering tumor growth, as indicated in Table 2(Tab. 2) (Zhang et al., 2009[199]; Mikutis et al., 2013[110]; Franceschin et al., 2014[55]; Tawani and Kumar, 2015[171]; Mondal et al., 2016[114]; Dwivedi et al., 2021[49]; Sanchez-Martin et al., 2022[151]). 

In summary, these polyphenolic compounds exemplify the diverse therapeutic possibilities of polyphenols derived from natural sources. Considering the significant evidence of their G4-mediated actions, polyphenols offer a compelling chance to utilize these readily available plant compounds for cancer therapy. However, challenges remain in enhancing their specificity, stability, and practical application in clinical environments, necessitating additional research to fully realize their potential as effective therapeutic agents.

### Dietary polyphenols and their potential role in epigenetic regulation through G4 binding

**Figure 6:**
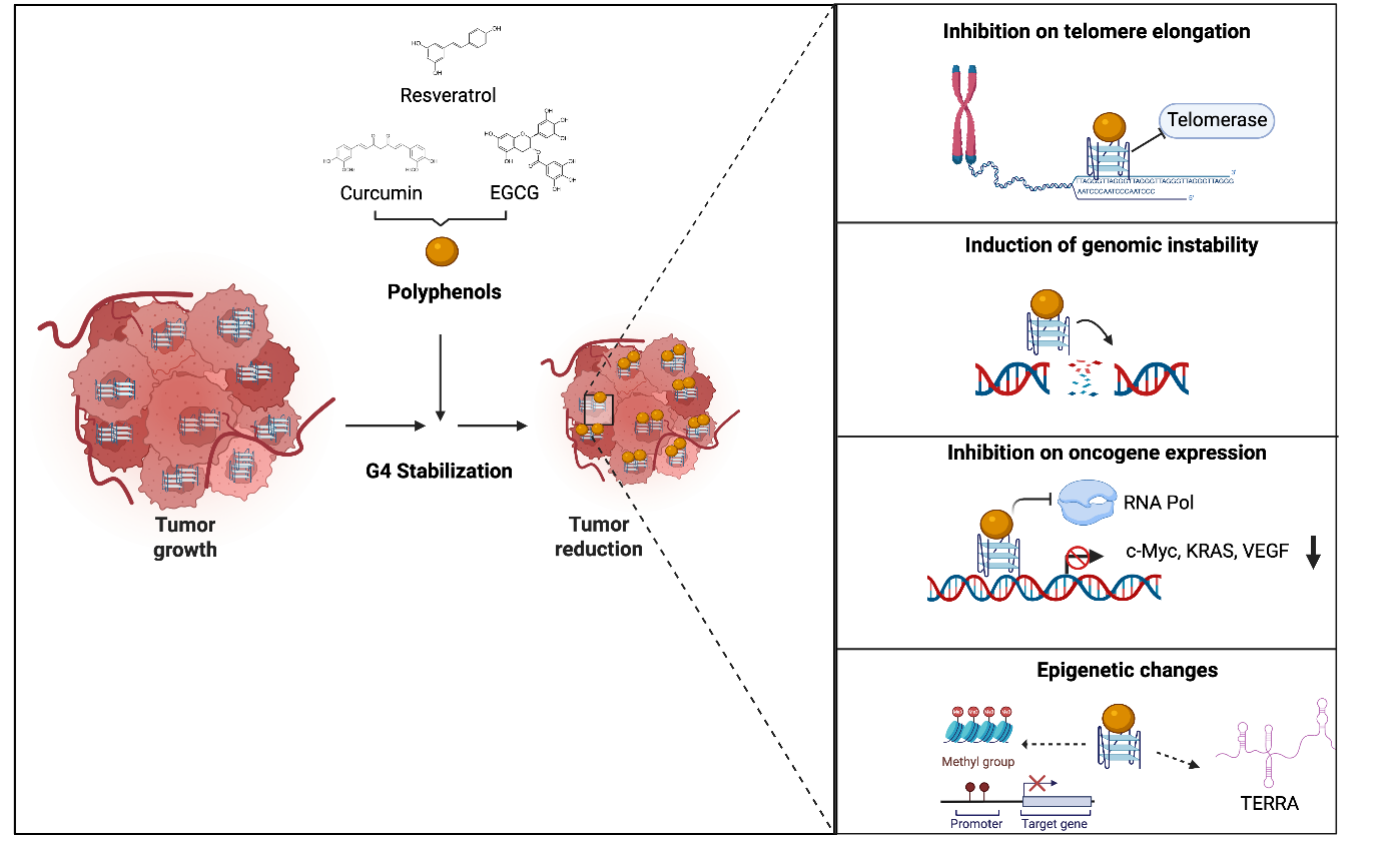
Role of Polyphenols in G-quadruplex (G4) Stabilization in Cancer. This schematic illustrates the role of G4 structures in cancer cells and the modulatory effects of polyphenols. G4 structures are elevated in tumor cells, contributing to tumor growth. Polyphenols stabilize G4 structures, which reduce tumor progression. The inset panels highlight specific effects of polyphenol-induced G4 stabilization, including inhibition of telomere elongation, induction of genomic instability, suppression of oncogene expression (e.g., *c-Myc, KRAS, c-KIT*), and epigenetic modifications such as promoter methylation alterations and regulation of non-coding RNA (TERRA).

Omics approaches, including genomics, transcriptomics, proteomics, and metabolomics, have uncovered the molecular mechanisms underlying diseases. Epigenetic mechanisms modify gene expression without altering the genetic sequence, involving changes in chromatin such as DNA methylation, histone modifications, nuclear structure, and the expression of non-coding RNAs (Yu et al., 2024[196]).G4s are epigenetic features responsive to stimuli, influencing transcriptomic changes by modulating gene expression in regulatory regions like enhancers and promoters (Halder et al., 2012[65]). Environmental signals, including food chemicals, affect G4 folding and stability, directly or indirectly, and G4s interact with other epigenetic alterations, shaping transcriptional outputs (François et al., 2015[57]; Reina and Cavalieri, 2020[141]). Epidrugs aim to restore the activity of silenced tumor suppressor genes by targeting enzymes such as DNMTs and HDACs to revert the abnormal epigenetic profiles found in cancer cells, and they hold potential as both biomarkers and personalized therapy options (Miranda Furtado et al., 2019[111]). Polyphenols represent promising candidates for epigenetic interventions, especially in cancer prevention and treatment, by influencing DNA methylation, histone changes, and the expression of non-coding RNAs. However, their specific mechanisms of action are not fully understood (Link et al., 2010[99]; Jayasinghe et al., 2016[78]). Bioactive compounds such as curcumin, resveratrol, EGCG, quercetin, genistein, and kaempferol influence epigenetic processes by reactivating tumor suppressor genes that have been silenced through hypermethylation. In myeloma cells, curcumin reduces mTOR levels via hypermethylation at the promoter region, which increases the expression of DNMT3a and DNMT3b (Chen et al., 2019[28]). In head and neck squamous cell carcinoma (HNSCC), EGCG lowers DNA hypermethylation, reduces DNMT activity, and increases tumor suppressor genes, suppressing tumor growth (Agarwal et al., 2023[2]). In colorectal cancer, kaempferol interacts with DNMT1 to reactivate DACT2, which hinders the Wnt/β-catenin signaling pathway and decreases tumor burden (Lu et al., 2018[102]). When combined with TSA, quercetin increases the p300 levels and promotes histone acetylation, thereby enhancing apoptosis in lung cancer cells (Chuang et al., 2019[32]). The combination of genistein and sulforaphane inhibits HDACs and histone methyltransferases, reducing the viability of breast cancer cells and decreasing tumor size (Paul et al., 2018[130]). Resveratrol enhances HDACi lethality in AML cells by inhibiting NF-κB via SIRT1 and stabilizing G4-related processes (Yaseen et al., 2012[194]). EGCG influences lncRNAs and mRNAs in lung cancer cells, affecting cell cycle regulation (Hu et al., 2019[71]). Curcumin alters miRNAs in ovarian cancer cells, enhancing sensitivity to cisplatin and hindering oncogenesis (Ravindran et al., 2023[139]). Resveratrol enhances the stability of TERRA G4 and markers of telomeric heterochromatin, promoting apoptosis in myeloma cells (Cusanelli and Chartrand, 2015[35]). Although the epigenetic modulation of polyphenols is evident, their involvement in G4 stabilization necessitates further investigation. The ability of resveratrol to stabilize TERRA G4s in conjunction with epigenetic regulation implies a connection, and additional studies are required to examine how polyphenols might restore gene expression in cancer through G4 stabilization and epigenetic effects (Figure 6(Fig. 6)).

Therefore, polyphenols are becoming recognized as promising compounds in the battle against cancer, affecting epigenetic processes and possibly interacting with G4 structures. Although the exact relationship between polyphenols and G4s requires further investigation, their therapeutic promise is becoming more evident, presenting a natural and innovative alternative for cancer therapy. Additional research will certainly yield a more profound understanding of these fascinating substances and their role in restoring proper gene expression.

## Clinical Relevance and Future Directions

Natural products and their related drugs compose approximately 35 % of the worldwide pharmaceutical industry, with 85 % of the global population depending on traditional medicine and 60 % of drugs in developed countries being natural or derived from natural sources (Mathur and Hoskins, 2017[107]; Najmi et al., 2022[119]; Nasim et al., 2022[120]). Despite promising bioactivities reported for polyphenols, they require safety and efficacy evaluations to minimize possible toxicity. Detailed preclinical and clinical research, including those listed on ClinicalTrials.gov, examining their safety, bioavailability, and effectiveness in preventing and treating cancer is presented in Table 3(Tab. 3). Curcumin has been shown to be safe and exhibit modest effects in colorectal cancer (Kanai et al., 2011[82]). Nevertheless, a Phase II trial on endometrial carcinoma did not reveal significant anti-inflammatory or immunomodulatory effects, although there was a noted improvement in quality of life (Tuyaerts et al., 2019[178]). The results concerning resveratrol in prostate cancer are mixed: one study suggested it delayed recurrence by extending the prostate-specific antigen (PSA) doubling time by 5.3 months (Paller et al., 2015[124]), while another study found no effect on prostate volume or PSA levels, raising doubts about its efficacy (Kjær et al., 2015[88]). The instability of polyphenols in various physiological conditions and the interindividual variability-arising from variations in metabolism, absorption, and interactions with dietary constituents-make their application and use complex. Only 5-10 % of the polyphenols consumed are absorbed in the small intestine; the remainder is metabolized by gut microbiota in the large intestine into more easily absorbable low-molecular-weight compounds that then undergo phase I and II metabolism (Farhan, 2023[51]; Li et al., 2023[96]). Nanotechnology can potentially enhance polyphenols' therapeutic effects by improving bioavailability and facilitating targeted delivery (Kim et al., 2023[85]). Nonetheless, polyphenols might interfere with drug metabolism by inhibiting cytochrome P450 enzymes and interacting with transporters like P-glycoprotein, which could lead to adverse effects (Duda-Chodak and Tarko, 2023[48]). Unlike epidrugs such as DNMT inhibitors (like 5-azacytidine) and HDAC inhibitors (such as vorinostat), which reliably reverse epigenetic alterations (Umehara, 2022[180]), the multitarget nature of polyphenols enables them to influence various pathways, potentially reducing resistance and toxicity when combined with chemotherapy (Herranz-López et al., 2018[69]). These complex factors are challenging for drug screening yet simultaneously provide prospects for synergistic effects in combination therapies. While there are challenges concerning stability, absorption, and drug interactions, the potential of polyphenols to affect various pathways, particularly in epigenetic regulation, presents a hopeful opportunity for innovation. As clinical trials progress, the transition from conventional remedies to effective treatments appears very encouraging, with polyphenols poised to be integral in the future of cancer treatment.

## Concluding Remarks

G-quadruplexes (G4s) are attractive targets in anticancer drug design. The presence of G4 structures in oncogenic promoters and telomeres indicates their potential role in regulating gene expression, making them critical in cancer biology. G4s structures play a crucial role in epigenetic regulatory mechanisms, influencing DNA methylation, histone modifications, and nuclear architecture. Their dysregulation is directly linked to various diseases, particularly cancer, where they drive gene expression changes that facilitate malignant transformation. The study of G4s deepens our understanding of gene regulation complexity and unveils new potential targets for therapeutic intervention, especially in cancer treatment. Natural products such as dietary polyphenols and their derivatives are invaluable resources for finding and developing new drugs. They could lead to safer anticancer medicines with strong selectivity for G-quadruplexes over duplex DNA, potentially leading to lower toxicity in vivo and minimizing side effects associated with traditional treatments. Polyphenols have significant potential as anticancer agents due to their ability to regulate epigenetic pathways and modulate gene expression involved in cancer progression; therefore, they could be developed into effective therapeutic interventions for cancer treatment. Although there is limited research on regulating G4s by polyphenols through an epigenetic mechanism, ample evidence shows that polyphenols regulate the epigenetic machinery. For example, it has been reported that compounds such as Curcumin, resveratrol, EGCG, quercetin, genistein, and kaempferol can regulate distinct epigenetic mechanisms, including DNMTs and HDAC enzymes. This can lead to the reactivation of tumor suppressor genes silenced by hypermethylation. Notably, studies have demonstrated that resveratrol can increase TERRA transcript expression and telomeric heterochromatin markers (H3K27me3 and H4K20me3), decreasing the proliferation of multiple myeloma cells. However, more studies are needed to fully harness polyphenols' therapeutic potential and better understand the molecular mechanisms by which they interact with G4 structures and their overall impact on cancer biology. Meanwhile, promoting a diet rich in polyphenols as a healthy eating habit may be linked to a lower cancer risk, highlighting the importance of dietary choices in cancer prevention strategies. 

## Declaration

### Authors contributions

MAMR, MHCR, and KGCL wrote and designed the original draft. AGC. and RFGL supervised, wrote, supervised, and validated the manuscript. All authors conceptualized the review and elaborated on the figures. All authors contributed to the article and approved the submitted version.

### Conflict of interest

The authors have no conflict of interest to declare.

### Acknowledgments

Author Marco Antonio Meraz-Rodriguez is grateful to UNAM “Programa de Maestría y Doctorado en Ciencias Bioquímicas” and SECIHTI (CVU:825532, no. 801922). Author Manuel Humberto Cháirez‐Ramírez is very thankful to the UNAM Postdoctoral Program (POSDOC).

## Figures and Tables

**Table 1:**
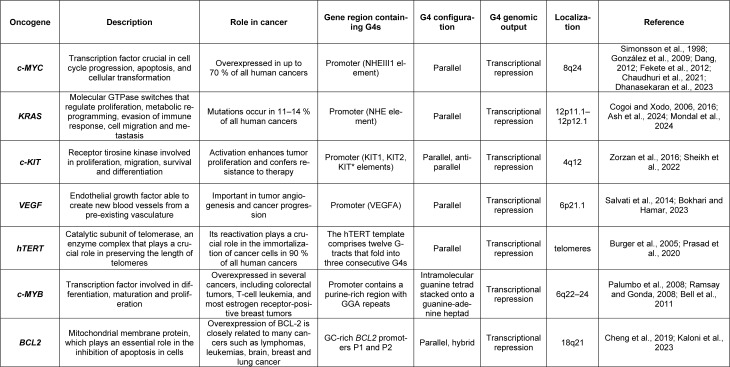
Reported oncogenes with G4 structures

**Table 2:**
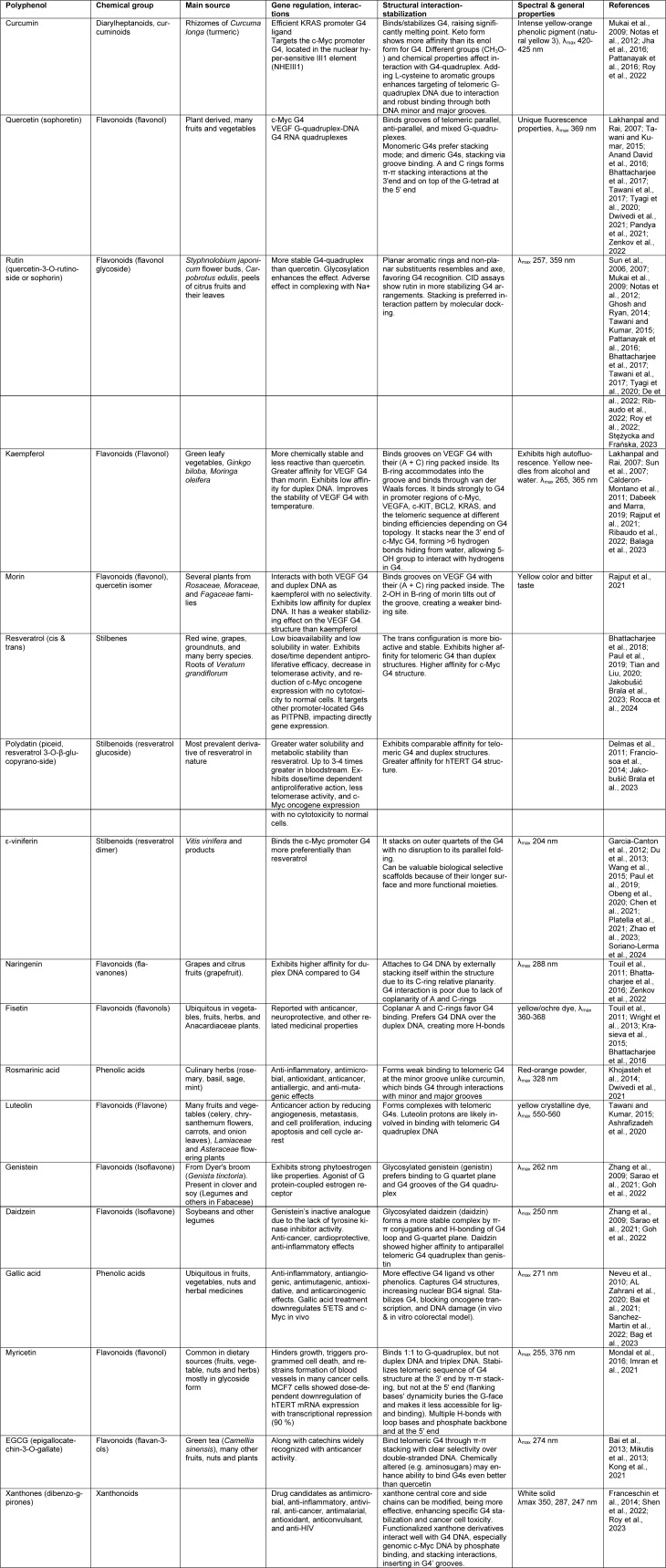
Chemical, physical, and biological properties of polyphenols

**Table 3:**
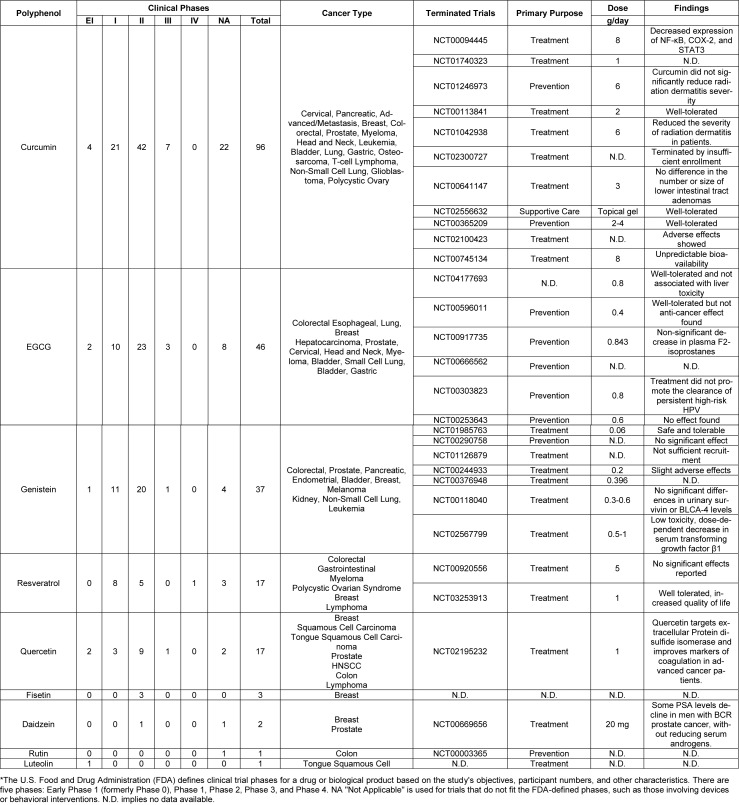
Overview of clinical trials registered in clinicaltrials.gov database using polyphenols for cancer therapies
